# Supplementary material for: DMRforPairs: identifying Differentially Methylated Regions between unique samples using array based methylation profiles
Source: BMC Bioinformatics. 2014 May 15;15:141. doi: 10.1186/1471-2105-15-141 (PMC4046028; doi:10.1186/1471-2105-15-141)
Supplement: Additional file 2 — DMRforPairs output for the comparison of A431-MCF7 and NA17018-NA17105. Please start from the HTML files in each folder. Available via the BMC Bioinformatics website. [file 1471-2105-15-141-S2.zip › 1394847754114233_MOESM2_ESM/A431_MCF7/figures/25746.pdf]

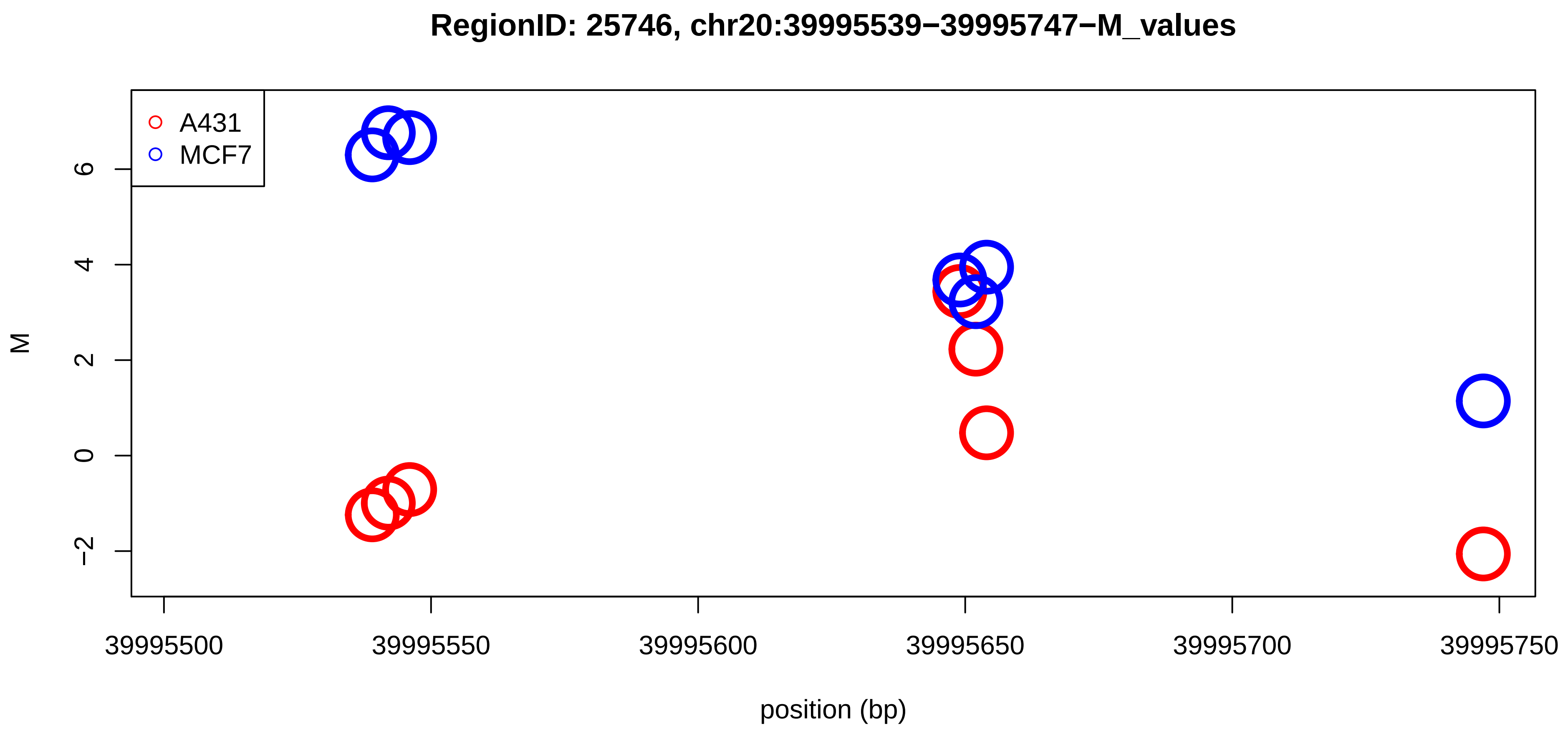

RegionID: 25746, chr20:39995539−39995747−M_values
A431
MCF7
M
−2
0
2
4
6
39995500
39995550
39995600
39995650
39995700
39995750
position (bp)

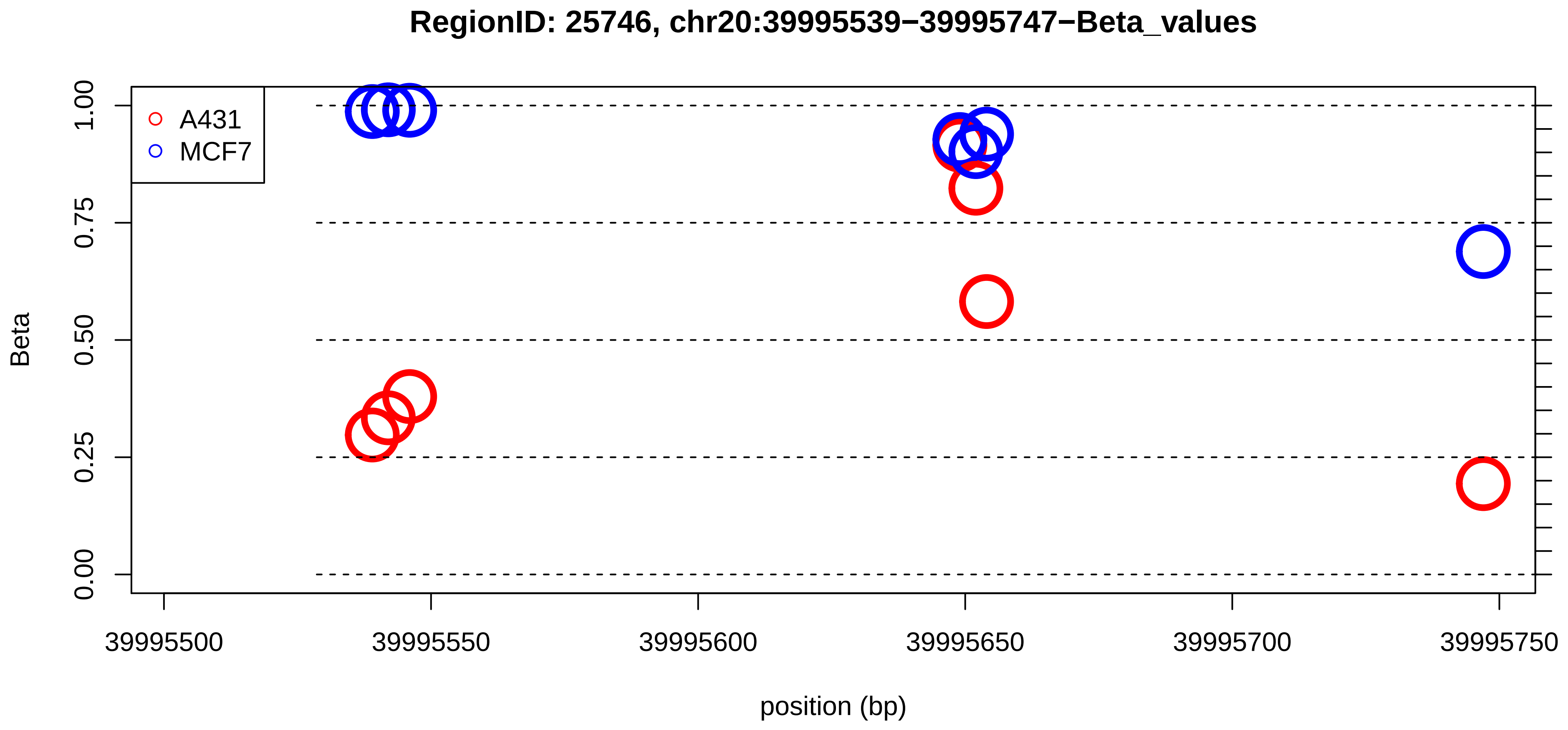

RegionID: 25746, chr20:39995539−39995747−Beta_values
A431
MCF7
Beta
0.00
0.25
0.50
0.75
1.00
39995500
39995550
39995600
39995650
39995700
39995750
position (bp)

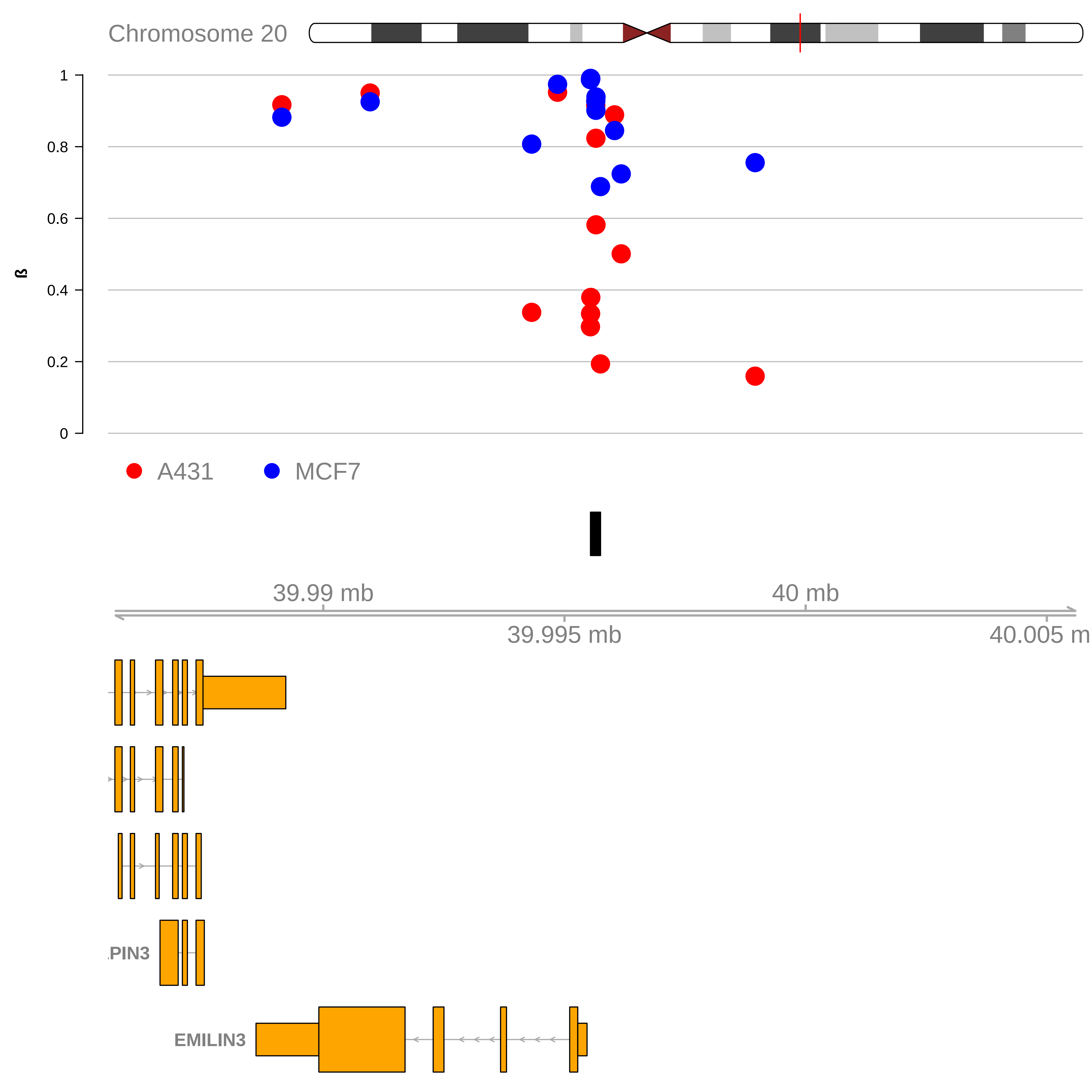

Chromosome 20
1
0.8
0.6
0.4
0.2
0
ß
A431
MCF7
39.99 mb
40 mb
39.995 mb
40.005 m
.PIN3
EMILIN3
